# Application of phase-conjugate beams in beam correction and underwater optical wireless communication subject to surface wave turbulence

**DOI:** 10.1007/s12200-022-00039-y

**Published:** 2022-09-14

**Authors:** Qi Li, Xiuhua Yuan, Feng Zhou, Zeyu Zhou, Wujie Liu

**Affiliations:** 1 School of Optical and Electronic Information, Huazhong University of Science and Technology, Wuhan, 430074 China; 2 Key Laboratory of Hunan Province On Information Photonics and Freespace Optical Communications, School of Information Science and Engineering, Hunan Institute of Science and Technology, Yueyang, 414006 China

**Keywords:** Phase-conjugate beam, Underwater optical wireless communication (UOWC), Degenerate four-wave mixing (DFWM), Surface wave turbulence

## Abstract

Water surface wave turbulence is one of the factors affecting the performances of underwater optical wireless communication (UOWC) systems. In our research, a phase-conjugate beam was used to correct the beam distortion and enhance the communication performances when a system is subject to surface wave turbulence. The phase-conjugate beam was generated by a phase-conjugate mirror (PCM), and a turbulence generator was used to generate surface wave turbulence in the experiment. We calculated the beam centroid distribution and the results showed that the phase-conjugate beam had a better propagation performance than the distorted beam at the different water depths. The root mean square (RMS) of the beam centroid for the phase-conjugate beam was 11 times less than that for the distorted beam, which meant that the phase-conjugate beam could effectively correct the beam drift. We further investigated the scintillation index and the signal-to-noise ratio (SNR); the results showed that the phase-conjugate beam was able to reduce the scintillation and an obvious improvement in SNR could be obtained. This research has the potential to be applied in UWC.

**Graphical Abstract:**

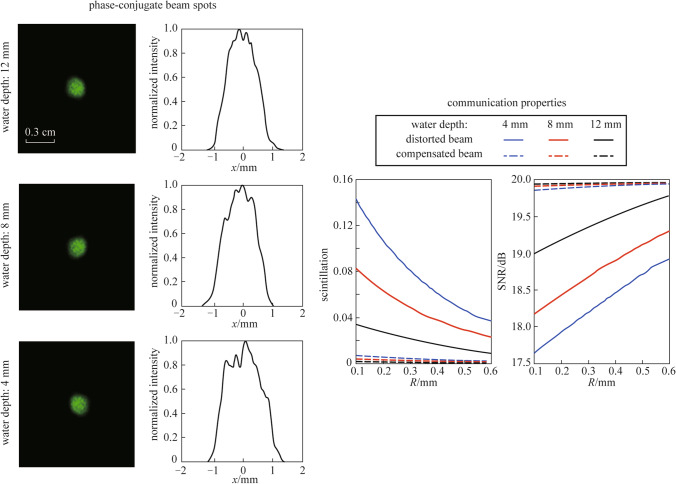

## Introduction

The research of ocean exploration has long been extensively concerned, and theoretical studies of underwater optical wireless communication (UOWC) technology have been reported [1–4]. In 1963, the scattering of light in the sea was investigated, and it was found that seawater exhibited a low absorption within the wavelengths range from 450 to 550 nm [5]. Due to the existence of this propagation “window”, several high-speed UOWC systems with blue-green light beams were designed and investigated [6–10]. However, when UOWC systems are installed close to the sea surface, the beam distortion from surface wave turbulence becomes a problem that cannot be ignored.

Surface wave turbulence can be described by weak-turbulence theory [11–13], and the beam centroid drift caused by the turbulence is the most significant disturbance for the UOWC systems [14–17]. After the laser beam propagates through the turbulence, the random refractive index leads to a phase distortion of the beam, which causes a beam centroid drift [11]. The phase distortion of beams can be compensated by a phase-conjugate beam [18, 19]. Thus, using a phase-conjugate beam to correct the beam drift caused by surface wave turbulence has been a viable solution. A phase-conjugate beam of blue-green beam has been efficiently obtained by a degenerate four-wave mixing (DFWM) method, where azo dyes-doped polymer was used as the nonlinear optical (NLO) media [20–23], thus pave the way for correction of beam distortion under the influence of surface wave turbulence.

In this work, a phase-conjugate beam generated by a phase-conjugate mirror (PCM) is used to correct the beam distortion caused by the surface wave turbulence, and the scintillation index and the signal-to-noise ratio (SNR) are evaluated. The distributions of centroids of the distorted beam at different water depths are calculated, and the distributions exhibit a Gaussian distribution. The centroid distributions and the root mean square (RMS) values of the source beam, the distorted beam, and the phase-conjugate beam are investigated, and the results show that the phase-conjugate beam is able to correct the beam drift caused by the surface wave turbulence. We further calculate the scintillation index and the SNR. For all the three cases investigated in the experiment where the water depth is 4, 8, and 12 mm, the phase-conjugate beam has lower scintillation indexes and higher SNRs than the distorted beam. The results show that using a phase-conjugate beam is able to improve the communication performances in a UOWC system.

## Concept description

### Generation of phase-conjugate beam

**Fig. 1:**
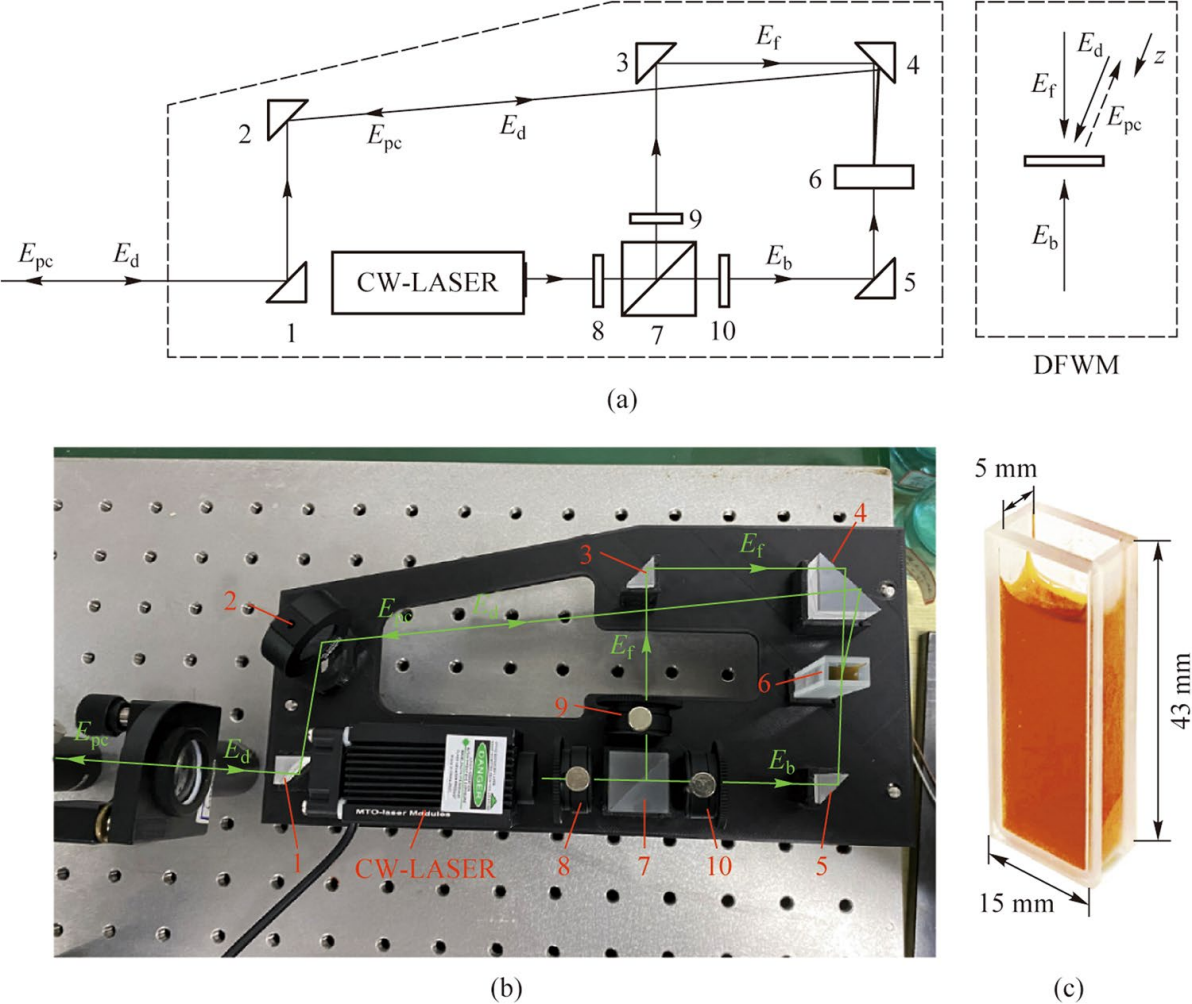
**a** Schematic of the PCM. ${E}_{\mathrm{f}}$, forward pump beam; ${E}_{\mathrm{b}}$, backward pump beam; ${E}_{\mathrm{d}}$, distorted beam, and ${E}_{\mathrm{pc}}$, phase-conjugate beam; CW-laser is a 532-nm CW laser; 1 − 5 are mirrors; 6 is the NLO medium; 7 is PBS; 8 − 10 are HWP. **b** Experimental setup for the PCM. **c** NLO medium in a cuvette

In this experiment, a PCM based on DFWM was designed, and the experimental setup is shown in Fig. 1a, b. The pump laser source was a 532-nm CW-laser with the output power of 50 mW. The beam was split into two branches of the equal intensity by the combination of a half-wave plate (HWP) (8) and a polarization beam splitter (PBS) (7); the reflected one was employed as the forward pump beam, while the transmitted one as the backward pump beam. The polarization of the forward and the backward pump beam were adjusted to be in the same direction by the HWP (9) and (10) respectively. The distorted beam denoted by the electric filed intensity ${E}_{\mathrm{d}}$ was directed by mirrors (1), (2), and (4), and then injected into the NLO medium (6). The NLO medium was prepared as follows. Methyl orange powder was added to polyvinyl alcohol solution and mixed using a water bath at 80 °C. After cooling down to room temperature, the NLO medium was contained in a cuvette (Fig. 1c) with an optical path of 0.5 mm.

DFWM is a third order nonlinear optical effect. Under optical pumping, the molecule structure of the methyl orange will change from trans-form to cis-form, which leads to a change of the electric polarization $P$. As can be seen in Fig. 1a, the forward pump beam and the backward pump beam, denoted by electric field ${E}_{\mathrm{f}}$ and ${E}_{\mathrm{b}}$ respectively, are counter-propagated. While the distorted beam denoted by ${E}_{\mathrm{d}}$ propagates along *z* axis and enters the NLO medium with a small angle to ${E}_{\mathrm{f}}$. The wave vectors of ${E}_{\mathrm{f}}$, ${E}_{\mathrm{b}}$, ${E}_{\mathrm{d}}$, and the phase-conjugate beam denoted by ${E}_{\mathrm{pc}}$ can be represented as ${{\varvec{k}}}_{\mathrm{f}}$, ${{\varvec{k}}}_{\mathrm{b}}$, ${{\varvec{k}}}_{\mathrm{d}}$, and ${{\varvec{k}}}_{\mathrm{pc}}$, respectively. The nonlinear polarization for the generation of phase-conjugate beam can be given by [18]

1 $${P}^{\left(3\right)}\left({\varvec{r}},t\right)=6{\varepsilon }_{0}{\chi }_{\mathrm{eff}}^{\left(3\right)}{E}_{\mathrm{f}}{E}_{\mathrm{b}}{E}_{\mathrm{d}}^{*}\left({\varvec{r}}\right){\mathrm{e}}^{-\mathrm{i}\left(\omega t-{{\varvec{k}}}_{\mathrm{pc}}z\right)}+c.c.,$$

where ${\varvec{r}}$ is the 2D position vector in the plane perpendicular to the propagation direction, $\omega$ is the angular frequency, *t* is the time, ${\varepsilon }_{0}$ is the permittivity in vacuum, ${\chi }_{\mathrm{eff}}^{\left(3\right)}$ is the effective third-order nonlinear susceptibility, and * is the complex conjugate. ${P}^{\left(3\right)}$ of Eq. (1) can be substituted into the wave equation ${\nabla }^{2}{E}_{\mathrm{pc}}-{\mu }_{0}\varepsilon \frac{{\partial }^{2}{E}_{\mathrm{pc}}}{\partial {t}^{2}}={\mu }_{0}\frac{{\partial }^{2}{P}^{\left(3\right)}}{\partial {t}^{2}}$, where $\varepsilon$ is the permittivity in the NLO medium, ${\mu }_{0}$ is permeability in vacuum. The expression for the light field of the phase-conjugate beam ${E}_{\mathrm{pc}}$ then becomes

2 $${E}_{\mathrm{pc}}\left({\varvec{r}}\right)=-\mathrm{i}\frac{{C}^{*}}{\left|C\right|}\mathrm{tan}\left(\left|C\right|L\right){E}_{\mathrm{d}}^{*}\left({\varvec{r}}\right),$$

where $C=-\frac{1}{{k}_{\mathrm{pc}}}3{\mu }_{0}{\varepsilon }_{0}{\chi }_{\mathrm{eff}}^{\left(3\right)}{E}_{\mathrm{f}}^{*}{E}_{\mathrm{b}}^{*}$, and $L$ is the optical path in the NLO medium.

To ensure a phase-matching of ${{\varvec{k}}}_{\mathrm{f}}+{{\varvec{k}}}_{\mathrm{b}}={{\varvec{k}}}_{\mathrm{d}}+{{\varvec{k}}}_{\mathrm{pc}}$, the propagation distances of the pump beams ${E}_{\mathrm{f}}$ and ${E}_{\mathrm{b}}$ from the PBS (7) to the NLO medium (6) were both set at 35 mm. To obtain a stable beam path, all the optical devices were precisely fixed to a custom-made base (Fig. 1b). The third-order nonlinear effect in the NLO medium led to the attenuation of two pump beams and the amplification of the distorted beam, and a phase-conjugate beam was generated in the opposite direction of the distorted beam. The intensity of the forward and backward pump beam were both 1 W/cm^2^. The phase conjugate reflectivity, which is defined as ${I}_{\mathrm{d}}/{I}_{\mathrm{pc}}$, where ${I}_{\mathrm{d}}$ and ${I}_{\mathrm{pc}}$ are the beam intensities of the distorted beam and the phase-conjugate beam, was 10% for the PCM used in the experiment.

### Experimental setup

**Fig. 2:**
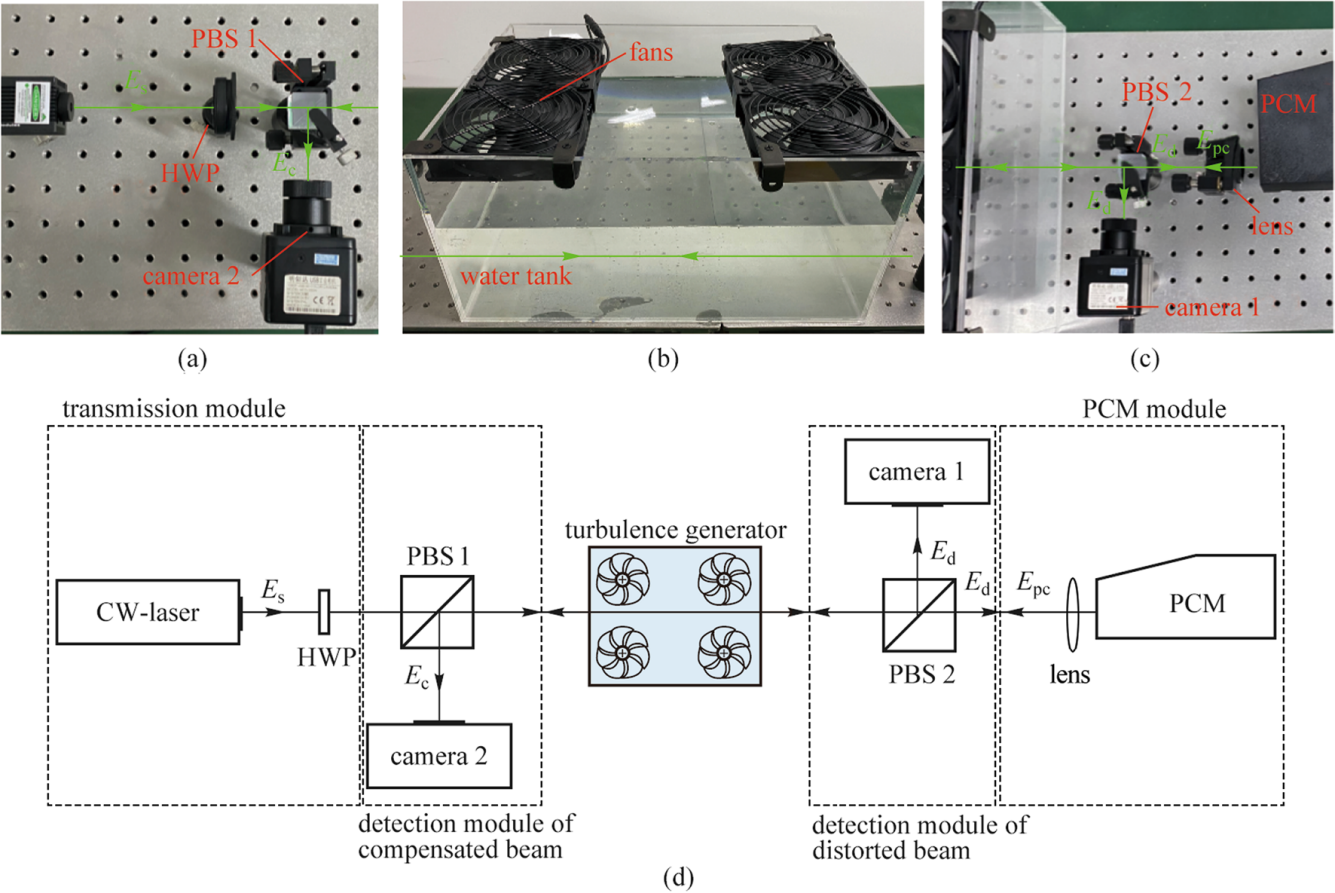
Experimental setup for beam drift correction caused by surface wave turbulence. ${E}_{\mathrm{s}}$, source beam, ${E}_{\mathrm{d}}$, distorted beam, ${E}_{\mathrm{pc}}$, phase-conjugate beam, and ${E}_{\mathrm{c}},$ compensated beam. **a** Transmission module and detection module of compensated beam. **b** Turbulence generator. **c** PCM module and detection module of distorted beam. **d** Schematic of the experimental setup

The pictures of the experimental setup are shown in Fig. 2 a–c. Figure 2a is the picture of the transmission module and the detection module of the compensated beam. The transmission module consisted of a 532-nm CW laser with output power of 3 mW and a HWP. Camera 2 and PBS 1 was used to collect the beam intensity distributions of the compensated beam. As can be seen in Fig. 2b, a water tank and four fans were used to generate surface wave turbulence. In this experiment, the surface wave turbulence was generated using pure water at 20 °C, because the phase-conjugate beam could only compensate for the phase distortions from random refractive media, and the uneven attenuation of the beam intensity caused by suspended scattering particles was not included in our investigation. The length, the width, and the height of the water tank were 45, 30, and 25 cm respectively, and the fans were set 5 cm above the water surface. In this experiment, the wind speed of the fans was 1.5 m/s. The maximum amplitude of the surface wave was measured to be 1.2 cm, and the oscillation frequency is about 0.7 Hz. Figure 2c is the picture of the PCM module and the detection module of the distorted beam. Camera 1 and PBS 2 was used to collect the beam intensity distributions of the distorted beam.

The experimental setup for beam drift correction under surface wave turbulence is shown in Fig. 2d. The source beam is generated by a laser, and the polarization of the source beam was adjusted in the same direction as the pump beams by the HWP. After propagating through the water tank, the distorted beam was then split by PBS 2. The reflected beam was recorded by camera 1, and the transmitted beam was focused by a lens (*f* = 500 mm) and injected into the PCM. The phase-conjugate beam generated by the PCM (denoted by ${E}_{\mathrm{pc}}$) propagated along the opposite direction of the distorted beam. After propagating through the water tank, the compensated beam denoted by ${E}_{\mathrm{c}}$ was reflected by PBS 1 and collected by camera 2.

## Results and discussion

### Centroid drift of beam spots

**Fig. 3:**
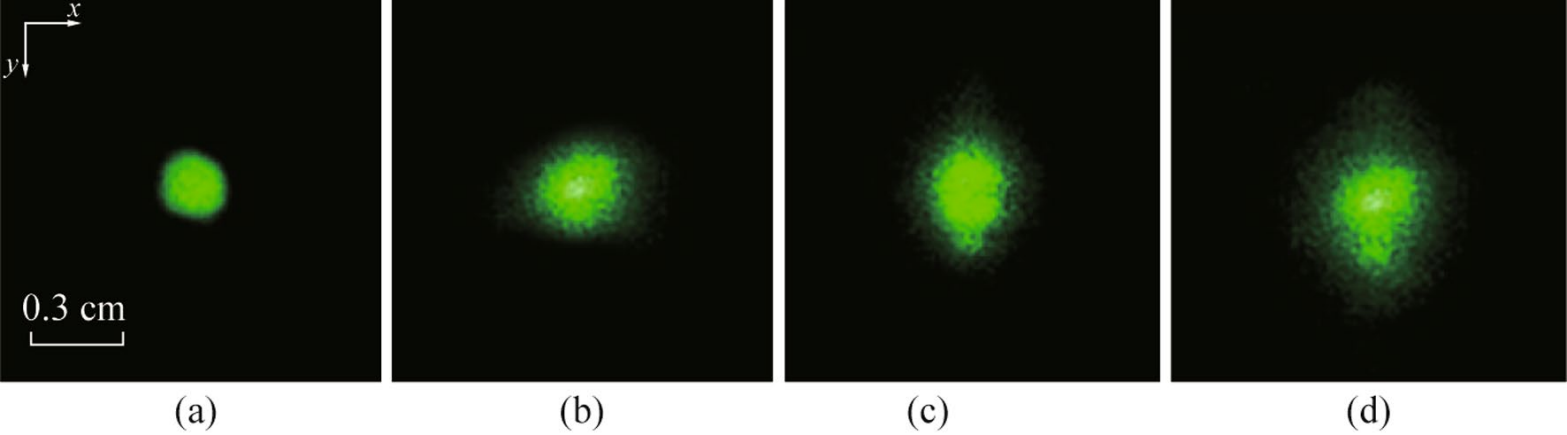
Typical image of laser spots. **a** Undistorted beam without surface wave turbulence. **b** Distorted beam at the water depth of 12 mm. **c** Distorted beam at the water depth of 8 mm. **d** Distorted beam at the water depth of 4 mm

In order to investigate the effect of surface wave turbulence on the under-water beam propagation, the optical path was set below the water surface. There are small bubbles in the water close to the water surface, and the water surface wave generated by the fans caused uneven pressure leading to random changes in the bubbles. When the beam propagated near the water surface, the refractive index fluctuations caused by bubbles leading to a beam drift. This phenomenon is mainly caused by phase distortion, so it can be corrected by the phase-conjugate beam. However, the phase-conjugate beam cannot compensate the beam distortion caused by the scattering phenomenon. In this paper, we do not discuss the compensation of scattering effect. The sampling speed of camera 1 and camera 2 was set to be 2 ms per frame. Figure 3a shows the laser spot after propagating through the water tank when the fans over the water surface are switched off. It can be seen that the beam spot is circular, and there is no significant beam distortion. We further investigated beam distortion under surface wave turbulence for different water depths. Figure 3b–d shows the beam spot at the water depths of 12, 8, and 4 mm respectively, and the depth of the beam in the water can be varied by adjusting the height of the optical module mounts. It is shown in Fig. 3b–d that the laser spots exhibited a distortion of intensity distribution.

**Fig. 4:**
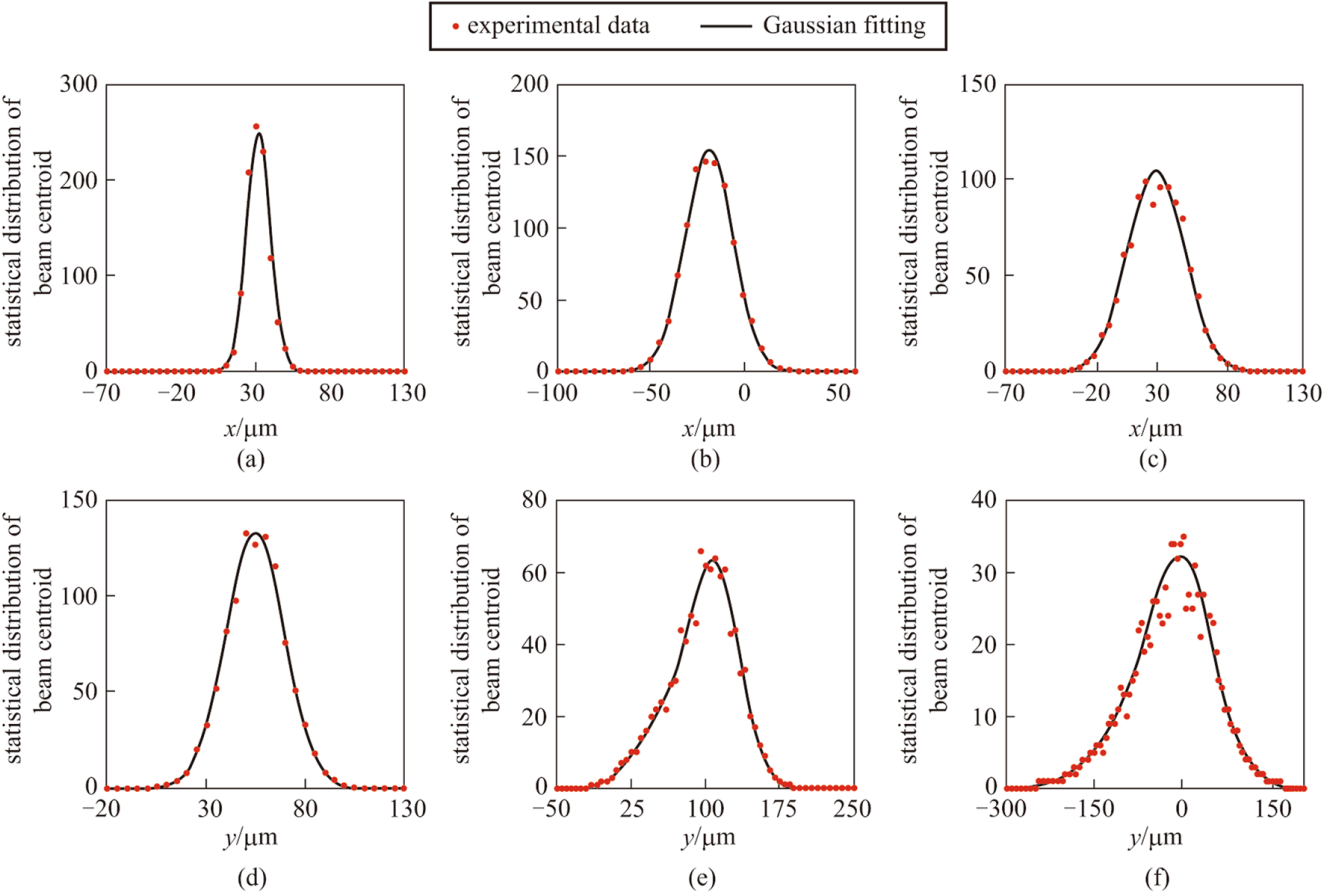
Statistical distribution of beam centroid with Gaussian fitting for the distorted beam. **a**–**c** The beam centroid distribution in the horizontal direction at water depth of 12, 8, and 4 mm respectively. **d**–**f** The beam centroid distribution in the vertical direction at water depth of 12, 8, and 4 mm respectively

To further understand the beam centroid distribution under the surface wave turbulence, the coordinates of the beam centroid $({x}_{n},{y}_{n})$ for the *n*th frame of image recorded by the camera were calculated as follows:

3 $${x}_{n}=\sum_{i}\sum_{j}{x}_{i}{I}_{n}({x}_{i},{y}_{j})/\sum_{i}\sum_{j}{I}_{n}({x}_{i},{y}_{j}),$$

4 $${y}_{n}=\sum_{i}\sum_{j}{y}_{j}{I}_{n}({x}_{i},{y}_{j})/\sum_{i}\sum_{j}{I}_{n}({x}_{i},{y}_{j}),$$

where *x* and *y* are the horizontal and vertical coordinates of the camera receiving plane respectively, ${I}_{n}({x}_{i},{y}_{j})$ is the beam intensity at camera pixel $(i,j)$ and $({x}_{i},{y}_{j})$ denotes the position of pixel $(i,j)$. The CMOS camera recorded 1000 images of the distorted beam spots at the water depths of 4, 8, and 12 mm respectively, and position of the beam centroids along horizontal and vertical directions were calculated. The camera center was set to be the origin of the *X–Y* coordinates. The center of the beam was hard to be adjusted to coincide with the center of the camera, so the statistical distributions of beam centroid were shifted on the axis. However, the position of the beam source and the cameras was static during every 1000 sampling. In Fig. 4a–c, the results for the statistical distributions of beam centroids in horizontal direction at different water depths were given and fitted by Gaussian curves. Figure 4d − f shows the statistical distributions of beam centroids in the vertical direction, which was fitted by bimodal Gaussian curves. As the water depth decreased, the range of the centroid distribution enlarged, which means that the beam closer to the water surface experienced stronger distortion. The air flow provided by the fans was mainly downward, and the consequent movement of the water was greater in the vertical direction than in the horizontal direction, leading to uneven turbulence. As a result, after propagating through the water tank, the beam exhibited a greater centroid drift in the vertical direction than the horizontal direction, as comparing Fig. 4a–c and d–f. Additional, the disturbance of the beam in the vertical direction is uneven, so the centroid distributions in the vertical direction were not symmetrical.

### Beam drift correction

**Fig. 5:**
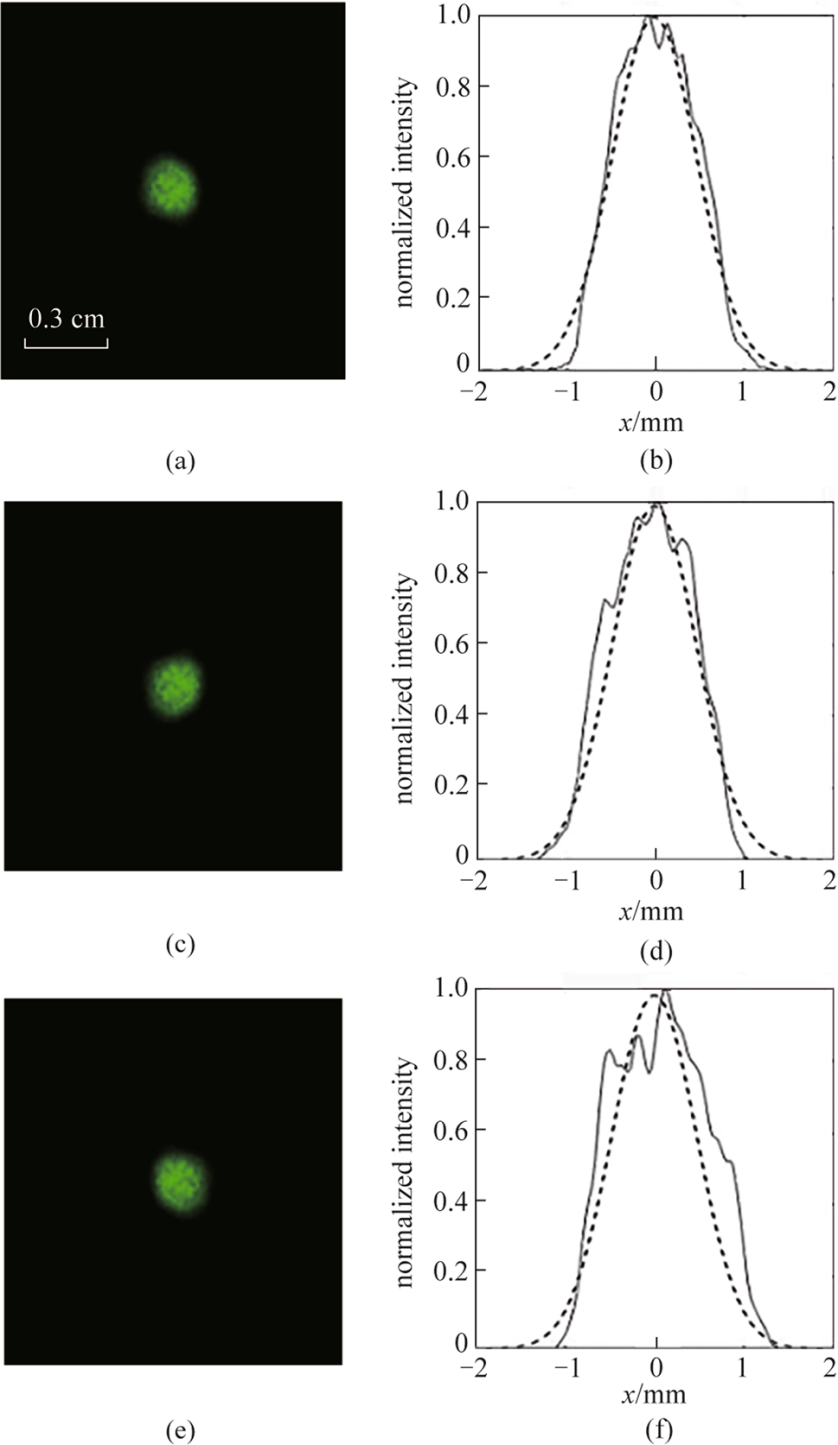
Laser spots and normalized intensity distributions of the compensated beam along the horizontal direction. **a**, **b** Water depth: 12 mm. **c**, **d** Water depth: 8 mm. **e**, **f** Water depth: 4 mm

The phase-conjugate beam was generated by the PCM and propagated along the opposite direction of the distorted beam. In this process, the turbulence could be regarded as a slowly varying disturbance. Theoretically, according to the theory of phase conjugation, the beam distortion caused by turbulence could be completely corrected after propagating back through the water tank. Figure 5 shows the laser spots and normalized intensity distributions of the compensated beams along the horizontal direction recorded by camera 2 at water depths of 12, 8, and 4 mm, respectively, and the dotted line is the normalized intensity distributions of the undistorted beam along the horizontal direction. The intensity distributions of the compensated beam were almost consistent with the undistorted beam at water depths of 12 and 8 mm. When the water depth decreased to 4 mm, the beam intensity distribution of the compensated beam had larger deviations from the Gaussian shape, which means the correction effect of phase conjugation was weakened.

**Fig. 6:**
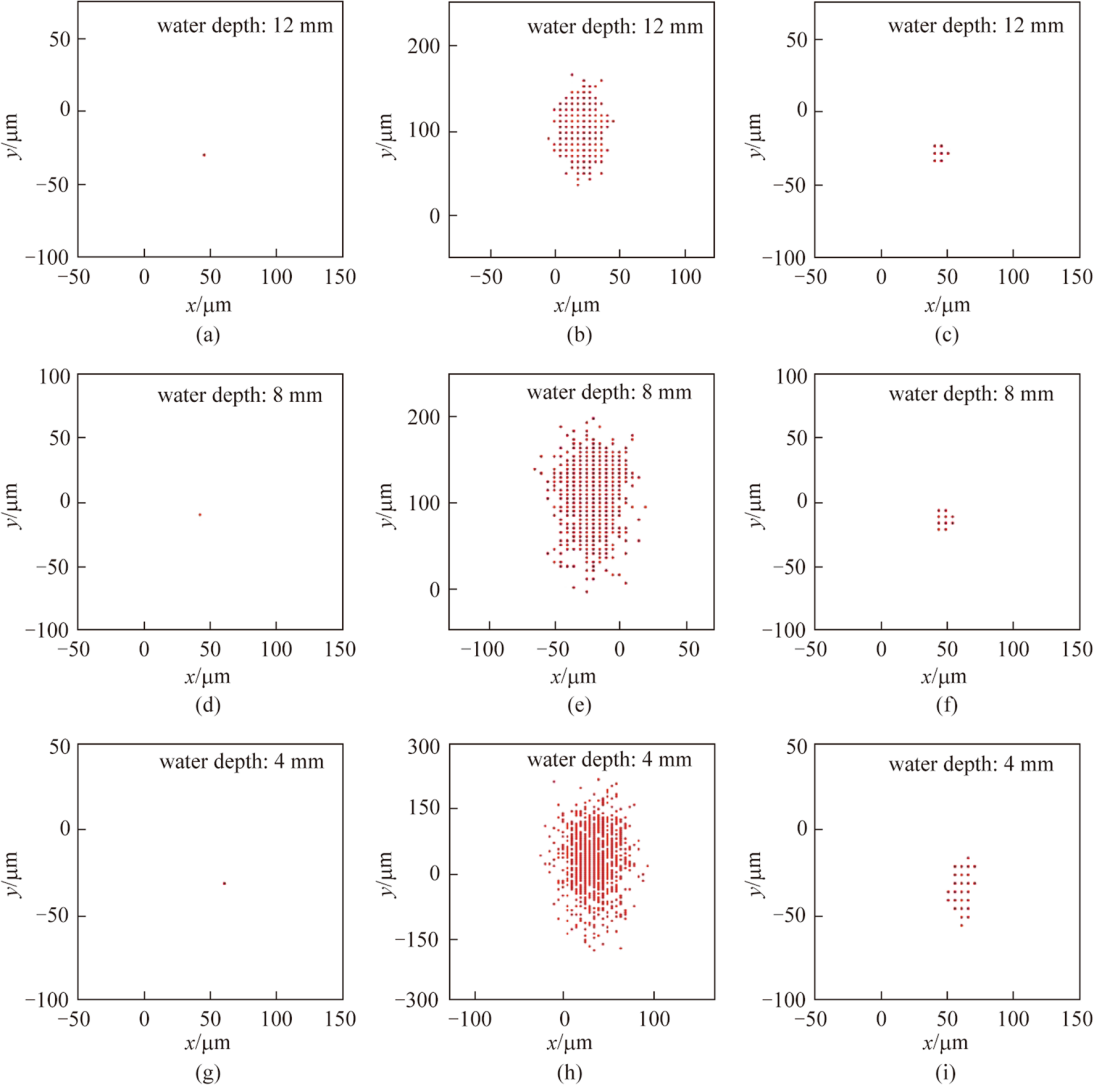
Centroid distributions of **a**, **d**, **g** undistorted beam, **b**, **e**, **h** distorted beam, and **c**, **f**, **i** compensated beam with the water depth of 12, 8, and 4 mm

To further evaluate the correction ability of the phase-conjugate beam, we calculated the centroid distributions of the undistorted beam and the compensated beam. When the fans were turned off, there was no turbulence in the water tank. Figure 6a, d, g show the centroid distribution of the undistorted beam at the water depths of 12, 8, and 4 mm respectively. It is obvious that the centroids of the undistorted beam did not change for different frames, which indicated that the undistorted beam exhibited no centroid drift after propagating through the stationary water tank.

When the fans were turned on, surface wave turbulence was generated. Figure 6b, e, h show the beam centroid distributions of the distorted beam at different water depths. With the decrease of the water depth, the distribution range of the centroid enlarged, which indicated that the distorted beam had larger beam drift when the propagation path is closer to the water surface. Figure 6c, f, i illustrate the centroid distributions of the compensated beam. In Fig. 6c, f, the centroid distribution range is significantly reduced compared to the cases of distorted beam, which is shown in Fig. 6b, e. The results demonstrated the correction effect due to phase-conjugation. In Fig. 6i, the centroid distribution range in the vertical direction is greater than that in the horizontal direction, and the centroid distribution range of the compensated beam at the water depth of 4 mm is seen to be greater than at the depths of 8 and 12 mm. This phenomenon can be explained by the DFWM process in the PCM. The drift of the distorted beam changed the angle of the beam entered the NLO medium, and decreased the generation quality of the phase-conjugate beam. Therefore, when the beam drift of the distorted beam is increased, the correction ability of the compensated beam is weakened.

By collecting the coordinates of centroid for each frame, the RMS of the beam centroid was calculated as follows:

5 $${\langle {r}_{\mathrm{c}}^{2}\rangle }^{1/2}={\left(\sum_{n=1}^{N}\frac{{\left({x}_{n}-\overline{x }\right)}^{2}+{\left({y}_{n}-\overline{y }\right)}^{2}}{N}\right)}^{1/2},$$

where $N$ is the number of frames, $(\overline{x }, \overline{y })$ is the average position of beam centroid for the *N* frames, expressed by

6 $$\overline{x }=\frac{1}{N}\sum_{n=1}^{N}{x}_{n},\space \overline{y }=\frac{1}{N}\sum_{n=1}^{N}{y}_{n}.$$

We recorded 1000 images for each water depth value, and the results of ${\langle {r}_{\mathrm{c}}^{2}\rangle }^{1/2}$ are presented in Table 1. The beam without turbulence had no shift at water depths of 12, 8, and 4 mm. The ${\langle {r}_{\mathrm{c}}^{2}\rangle }^{1/2}$ value of the distorted beam was 16.91 μm at water depth of 12 mm; this value increased to 72.93 μm when the water depth decreased to 4 mm. The compensated beam had a ${\langle {r}_{\mathrm{c}}^{2}\rangle }^{1/2}$ of 2.01 μm at 12 mm water depth. At the water depth of 4 mm, the ${\langle {r}_{\mathrm{c}}^{2}\rangle }^{1/2}$ value increased to 6.67 μm, which was about 11 times less than that of the distorted beam. The results show that the phase-conjugate beam had an excellent correction effect on the beam drift caused by surface wave turbulence.

**Table 1:** RMS of the beam centroid of the undistorted beam, the distorted beam and the compensated beam

Water depth/mm	RMS of the beam centroid ${\langle {r}_{\mathrm{c}}^{2}\rangle }^{1/2}$/μm		
	Undistorted beam	Distorted beam	Compensated beam
12	0	16.91	2.01
8	0	38.84	3.29
4	0	72.93	6.67

### Scintillation index and SNR

In UOWC system, beam scintillation is one of the main reasons for degrading the communication quality. The phase-conjugate beam can not only correct the beam shift caused by turbulence, but also reduce beam scintillation and improve communication quality. To further investigate the application potential of the phase-conjugate beam in UOWC system, we calculated the scintillation index and the SNR of the distorted beam and the compensated beam to evaluate the compensation effect of the beam intensity fluctuations and the communication quality by using phase-conjugate beams. The aperture-averaged scintillation index was defined as follows:

7 $${\sigma }_{\mathrm{I}}^{2}\left(R\right)=\frac{\langle {({\int }_{0}^{R}{\int }_{0}^{2\uppi }I(\overrightarrow{r})r\mathrm{d}r\mathrm{d}\theta )}^{2}\rangle }{{\langle {\int }_{0}^{R}{\int }_{0}^{2\uppi }I(\overrightarrow{r})r\mathrm{d}r\mathrm{d}\theta \rangle }^{2}}-1,$$

where, $I\left(\overrightarrow{r}\right)$ is the beam intensity distribution, *R* is the radius of camera aperture. With the scintillation index obtained, the SNR could be calculated using:

8 $$\langle SNR\rangle =\frac{{SNR}_{0}}{\sqrt{{\sigma }_{\mathrm{I}}^{2}\left(R\right){SNR}_{0}^{2}+{P}_{\mathrm{S}0}/\langle {P}_{\mathrm{S}}\rangle }},$$

where ${SNR}_{0}$ is the signal-to-noise ratio of the undistorted beam, which is assumed as 20 dB, ${P}_{\mathrm{S}0}$ is the signal power at the source plane, and $\langle {P}_{\mathrm{S}}\rangle$ is the average signal power at the receiver plane. In the experiment, the size of the camera aperture was constant, while the aperture-averaged scintillation index and SNR for different apertures are obtained by calculating the beam intensities within a circle of a specific radius.

**Fig. 7:**
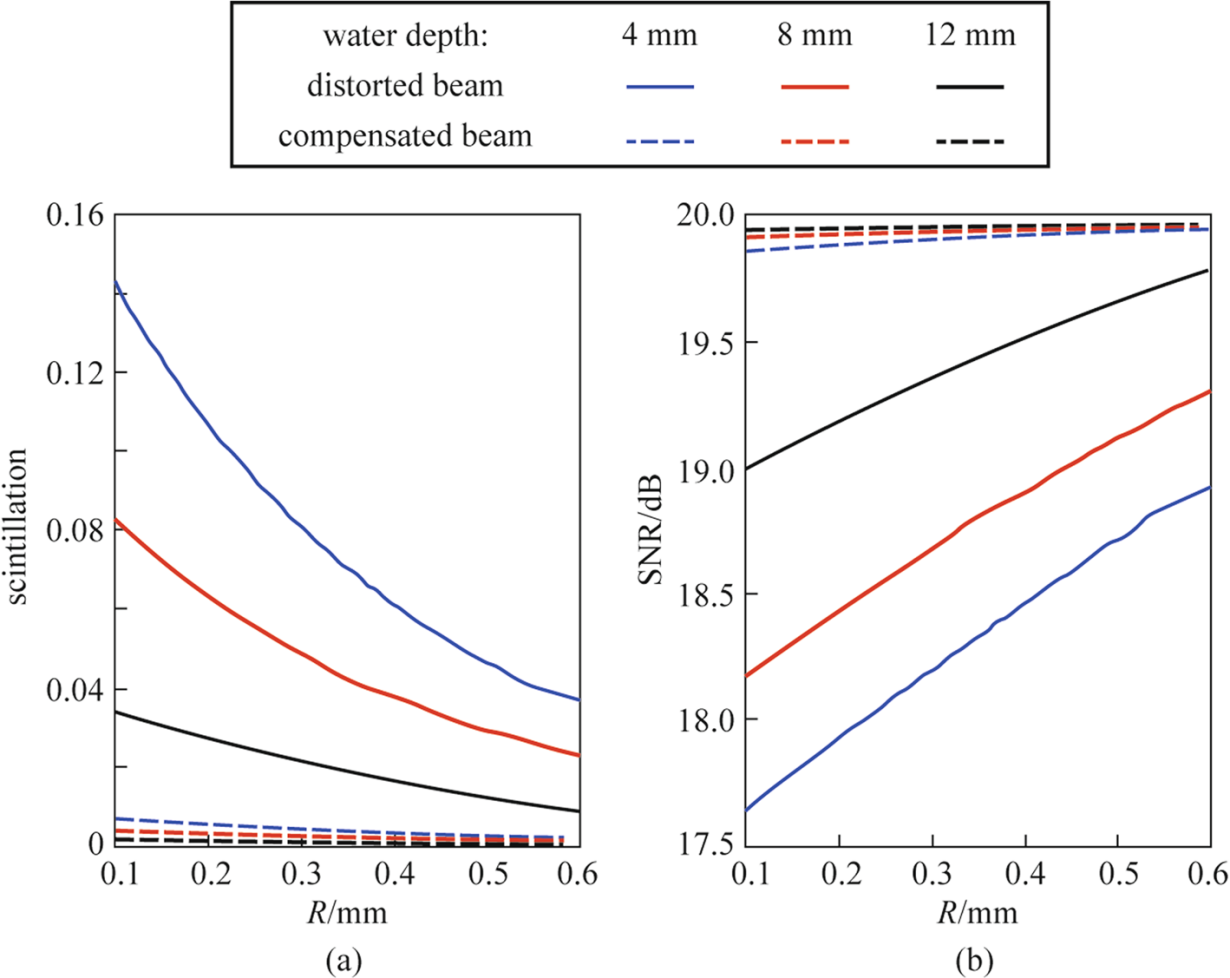
**a** Scintillation index and **b** SNR versus aperture radius *R* of the distorted beam and compensated beam at water depths of 4, 8, and 12 mm

The results of the scintillation index and the SNR are shown in Fig. 7. As can be seen in Fig. 7a, increasing the aperture radius led to a lower scintillation, which can be attributed to the larger averaging effect for larger apertures, according to Eq. (7) Besides, for the same aperture radius, the smaller the water depth, the higher the scintillation index. This behavior is due to the stronger turbulence for the small water depth. By comparing the scintillation index curve of the compensated beam and the distorted beam, it could be seen that the scintillation index of the compensated beam was significantly reduced. Figure 7b displays the SNR performance of the distorted beam and the compensated beam. At the water depth of 4 mm, the compensated beam had a SNR improvement of more than 2 dB over the distorted beam with the aperture radius of 0.1 mm. The results show that phase-conjugation provides an effective way to improve communication performance for UOWC systems.

## Conclusion

In this research, the phase-conjugate beam is used to correct the beam distortion caused by surface wave turbulence in UOWC systems. A PCM was built to generate the phase-conjugate beam by using the DFWM method. The intensity distribution of the distorted beams and compensated beams are recorded by CMOS cameras. The results show that the beam drift is larger for a water path closer to the water surface, and beam drift along the horizontal direction is greater than that along the vertical distribution. By comparing the centroid distribution of the distorted beam and the compensated beam, it can be seen that the beam drift caused by the surface wave turbulence in the phase-conjugate beam can be effectively corrected. In addition, correction effect in the horizontal direction is better than in the vertical direction. To quantify the correction effect of the phase-conjugate beam, the RMS of the beam centroid of the undistorted beam, the distorted beam, and the compensated beam are calculated and results show that the RMS of the beam centroid decreases from 72.93 (distorted beam) to 6.67 μm (compensated beam) at the water depth of 4 mm. To further evaluate the communication performance of the UOWC system under surface wave turbulence, the scintillation index and SNR of the distorted beam and the compensated beam are investigated. The results show that the compensated beam has a higher scintillation index and better SNR performance. Therefore, we conclude that phase-conjugation provides an effective way to improve the communication performances of UOWC systems.
